# Development of a Novel Anti-CD19 CAR Containing a Fully Human scFv and Three Costimulatory Domains

**DOI:** 10.3389/fonc.2021.802876

**Published:** 2022-01-18

**Authors:** Yupanun Wutti-in, Jatuporn Sujjitjoon, Nunghathai Sawasdee, Aussara Panya, Katesara Kongkla, Pornpimon Yuti, Petlada Yongpitakwattana, Chutamas Thepmalee, Mutita Junking, Thaweesak Chieochansin, Naravat Poungvarin, Montarop Yamabhai, Pa-thai Yenchitsomanus

**Affiliations:** ^1^ Graduate Program in Immunology, Department of Immunology, Faculty of Medicine Siriraj Hospital, Mahidol University, Bangkok, Thailand; ^2^ Siriraj Center of Research Excellence for Cancer Immunotherapy (SiCORE-CIT), Faculty of Medicine Siriraj Hospital, Mahidol University, Bangkok, Thailand; ^3^ Division of Molecular Medicine, Research Department, Faculty of Medicine Siriraj Hospital, Mahidol University, Bangkok, Thailand; ^4^ Department of Biology, Faculty of Science, Chiang Mai University, Chiang Mai, Thailand; ^5^ Division of Biochemistry, School of Medical Sciences, University of Phayao, Phayao, Thailand; ^6^ Department of Clinical Pathology, Faculty of Medicine Siriraj Hospital, Mahidol University, Bangkok, Thailand; ^7^ Molecular Biotechnology Laboratory, School of Biotechnology, Institute of Agricultural Technology, Suranaree University of Technology, Nakhon Ratchasima, Thailand

**Keywords:** adoptive T cell therapy, human single-chain variable fragment, scFv, anti-CD19 CAR T cells, B-cell malignancies

## Abstract

Second-generation anti-CD19-chimeric antigen receptor T cells (anti-CD19-CAR2 T cells) are effective for treating B-cell malignancies; however, anti-CD19-CAR2 T cells can induce human anti-mouse immune responses because anti-CD19 single-chain variable fragment (scFv) in the CAR molecules is derived from a murine FMC63 (mFMC63) monoclonal antibody. Consequently, the persistence of mFMC63-CAR2 T cells and their therapeutic efficiency in patients are decreased, which results in tumor relapse. In an attempt to remedy this shortcoming, we generated a new anti-CD19-CAR T cells containing fully human anti-CD19 scFv (Hu1E7-CAR4 T cells) to pre-clinically evaluate and compare with mFMC63-CAR4 T cells. The human anti-CD19 scFv (Hu1E7) was isolated from a human scFv phage display library and fused to the hinge region of CD8α, the transmembrane domain of CD28, three intracellular costimulatory domains (CD28, 4-1BB, and CD27), and a CD3ζ signaling domain (28BB27ζ). Compared to mFMC63-CAR2 T cells (BBζ) and mFMC63-CAR3 (BB27ζ), the mFMC63-CAR4 T cells (28BB27ζ) exerted superior anti-tumor activity against Raji (CD19^+^) target cell. The Hu1E7-CAR4 and mFMC63-CAR4 T cells demonstrated comparable cytotoxicity and proliferation. Interestingly, compared to mFMC63-CAR4 T cells, the Hu1E7-CAR4 T cells secreted lower levels of cytokines (IFN-γ and TNF-α), which may be due to the lower binding affinity of Hu1E7-CAR4 T cells. These findings demonstrated the successfulness in creation of a new CAR T cells containing a novel fully human-derived scFv specific to CD19^+^ cancer cells. *In vivo* studies are needed to further compare the anti-tumor efficacy and safety of Hu1E7-CAR4 T cells and mFMC63-CAR4 T cells.

## Introduction

Cellular immunotherapy using autologous T cells expressing chimeric antigen receptor (CAR) is a promising strategy as evidenced by CD19-CAR T cell therapy for B-cell malignancies (1–3). CAR is a synthetic receptor consisting of an antigen-binding domain, which is usually a single-chain variable fragment (scFv) derived from a monoclonal antibody, that is linked by a hinge or spacer to a transmembrane domain and an intracellular signaling motif from T cell receptor complex containing CD3 zeta (CD3ζ) and costimulatory signaling domain(s) (4). CAR T cells mediate tumor cell killing by the binding of scFv of CAR to a specific target-antigen on the cancer cell surface without peptide-antigen presentation on major-histocompatibility complex (MHC). Upon direct binding to the specific target-antigen, CAR T cells are activated *via* the functions of intracellular signaling domains (5). The U.S Food and Drug Administration (FDA) has approved four CD19-CAR T products for relapsed/refractory acute lymphoblastic leukemia (r/r ALL) (2), r/r large B-cell lymphoma (6, 7) and mantle cell lymphoma (8). Although objective clinical responses after CAR T treatment were observed and documented, adverse effects [e.g., cytokine release syndrome (CRS) and/or neurologic toxicity] and tumor relapse were reported (2, 9).The mechanism of CD19^+^ relapse is associated with poor T cell function and early CD19-CAR T cell disappearance (10). The previously approved CD19-CAR T products are based on murine-derived scFv (FMC63) (11) that is fused to second-generation CAR [CD28ζ (6, 8) or 4-1BBζ (2, 7)]. In pharmacological studies, CD28-based CAR elicited anti-tumor activity more rapidly than 4-1BB-based CAR T cells that persisted longer *in vivo* (12). Previous studies added the 4-1BB costimulatory signal to CD28 to develop a third-generation CAR (CD28-41BBζ), which increased the expansion potential of CAR T cells *in vivo* (13). However, the potential benefits of this third-generation CAR over the second-generation CAR have not yet been conclusively established (14). Previous preclinical studies using CD27-based CAR construct demonstrated efficient anti-tumor response (15) and prolonged survival of CAR T cells *in vivo* by upregulating the anti-apoptotic protein Bcl-XL (16). Clinical studies were also conducted using fourth-generation anti-CD19-CAR (4SCAR19) T cells containing a combination of three costimulatory domains, and these CAR T cells showed long-term high efficiency and safety in B-ALL patients (17). These reported findings suggest that a combination of several costimulatory domains in the CAR construct may improve the functional potential of CAR T cells, including anti-tumor activity, T cell expansion, and T cell persistence *in vivo*. Therefore, we developed a new fourth-generation CAR (CAR4) containing three costimulatory signaling domains (28BB27ζ). Anti-tumor efficiencies of the new CAR4 construct had been reported in others solid tumor including cholangiocarcinoma (18–20) and breast cancer (21).

The development of anti-CAR immune responses was reported to be a factor that causes lower persistence of CAR T cells in patients (10). The presence of non-human (murine) scFv might induce human anti-mouse antibody (HAMA) response, and thereby limit CAR T cell persistence in patients (22). Several studies attempted to develop humanized scFv to reduce the immunogenicity of CAR (23–26). A recent phase I clinical trial using humanized scFv anti-CD19-CAR (hCD19-CAR) T cells for r/r pediatric ALL (27) showed that 16.7% (4/24) of patients had no response (NR). Of the four children with NR, three of them had previously received murine anti-CD19-CAR T product (FMC63-CAR T) before infusion with hCD19-CAR T cells. This data suggests that although hCD19-CAR T cells could reduce the immunogenicity of the murine (m) CAR construct, the presence of some murine CDR sequences may lead to immune response and tumor relapse. Therefore, an anti-CD19-CAR construct containing fully human anti-CD19 scFv would be a solution for clinical use.

To overcome the challenge of anti-CAR immune responses and to improve the anti-tumor activity of CD19-specific CAR T cells, we created a new anti-CD19 CAR T cells containing fully human scFv isolated from a phage display library. The fourth-generation CAR (CAR4) containing three costimulatory signaling domains (28BB27ζ) that previously reported (18–21) was selected to generate the human anti-CD19 (Hu1E7) CAR4 T cells and murine anti-CD19 (mFMC63) CAR4 T cells. Both mFMC63 and Hu1E7 anti-CD19 CAR4 T cells were generated, evaluated, and compared for their cytolytic activities, proliferative capabilities, and cytokine production.

## Materials and Methods

### Cell Lines and Cell Culture Conditions

Human cervical carcinoma (HeLa) cells (ATCC Cat# CRL-7923, RRID : CVCL_0030), Human Embryonic Kidney (HEK) 293T cells (ATCC Cat# CRL-3216, RRID : CVCL_0063), and Lenti-X™ 293T cells (Takara Bio, Inc., Shiga, Japan) were maintained in Dulbecco’s Modified Eagle Medium (DMEM) (Gibco; Thermo Fisher Scientific, Waltham, MA, USA) supplemented with 10% heat-inactivated fetal bovine serum (FBS) and 100 μg/ml of penicillin/streptomycin (Sigma-Aldrich Corporation, St. Louis, MO, USA). Raji, which is a B lymphocyte cell line developed from human Burkitt’s lymphoma (ATCC Cat# CCL-86, RRID : CVCL_0511), and K562, which is a cell line derived from human chronic myelogenous leukemia (CML) (ATCC Cat# CCL-243, RRID : CVCL_0004), were maintained in culture with Roswell Park Memorial Institute (RPMI)-1640 supplemented with 10% FBS and 100 μg/ml of penicillin/streptomycin.

### Identification of Human Anti-CD19 scFv by Phage Biopanning

The human scFv phage display library (Yamo-I) prepared from 140 healthy donors with a moderate size of gene repertoire diversity (1.5x10^8^) (28) was used for screening of anti-CD19 scFv. Briefly, HeLa-CD19^+^ or HeLa-eYFP (to serve as an irrelevant protein) were plated and blocked with 3% (w/v) skimmed milk in phosphate-buffered saline (PBS). HeLa-eYFP cells were first incubated with the Yamo-I phage display library containing 10^11^-10^13^ phage particles at 37°C for 1 h. After five rounds of subtraction, the unbound phages were incubated with HeLa-CD19^+^ cells at 37°C for 2 h. The unbound phages were washed with PBS containing 0.1% Tween 20. The bound phages were eluted with 1 mg/mL trypsin in PBS and 50 mM glycine-HCl (pH 2.0), and then neutralized with 200 mM NaHPO_4_ (pH 7.5). The eluted phages were propagated in *Escherichia coli* TG1 cells with KM13 helper phage for a further round of selection, as previously described (29).

### Human Anti-CD19 scFv Binding Assay

HeLa-CD19^+^ or parental HeLa cells were collected and incubated with each soluble human anti-CD19 scFv secreted in the *E. coli* culture supernatant. Non-infected *E. coli* HB2151 culture supernatant was used as a negative control. Following two washes with 2% FBS-PBS, the cells were stained with monoclonal anti-His (HIS.H8) antibody (Thermo Fisher Scientific), and incubated at 4°C for 1 h. Alexa Fluor-488 labeled goat anti-mouse IgG antibody (Cat# A28175; Thermo Fisher Scientific) was added to the cells with subsequent incubation at 4°C for 30 minutes (min) in the dark. The cells were washed twice and analyzed by flow cytometry (BD Accuri™ C6 Plus) (BD Biosciences).

### Lentiviral CAR Construction

Second-generation CAR (CAR2), third-generation (CAR3) and fourth-generation CAR (CAR4) cassette genes were synthesized by Integrated DNA Technologies (Coralville, IA, USA). The CAR2 comprises DNA sequences encoding a hinge region and transmembrane of CD8, an intracellular signaling domain of 4-1BB (or CD137), and CD3 zeta (CD3ζ). The CAR3 contains DNA sequences encoding a hinge region and transmembrane domain of CD8, and intracellular signaling domains derived from 4-1BB, CD27, and CD3ζ. The CAR4 contains DNA sequences encoding a CD8 hinge region, a transmembrane domain of human CD28, and intracellular signaling domains derived from CD28, 4-1BB, CD27, and CD3ζ. These three constructs were cloned into a self-inactivation (SIN) lentiviral vector (pCDH.*EF1α*.SIN.WPRE, RRID : Addgene_71708). The sequence of anti-CD19 scFv derived from murine (mFMC63) (11) was cloned into the CAR2, CAR3, and CAR4 constructs. The sequence of human anti-CD19 scFv (Hu1E7) was cloned into the CAR4 lentiviral vector. The sequence of anti-GD2 scFv (Hu3F8)-CAR4 construct was used as an irrelevant CAR4 control. The sequence encoding c-Myc tag was added between scFv and the CD8 hinge region in all constructs for detection of CAR expression on the cell surface by anti-Myc antibody (Abcam Cat# ab1394, RRID : AB_300767) staining and flow cytometry analysis (**Supplementary Figures 1**, **2**). The correctness of all sequences of CAR constructs was verified by Sanger sequencing.

### Production of Lentiviral Particles

Lentiviral particles containing mFMC63-CAR2, mFMC63-CAR3, mFMC63-CAR4, Hu1E7-CAR4, or Hu3F8-CAR4 were packaged in Lenti-X™293T cells as described previously (18). See **Supplementary Material** for details.

### T Cell Isolation and Gene Transfer

The protocol for the use of human blood samples from healthy volunteer donors was approved by the Siriraj Institutional Review Board (COA number: Si 762/2016) of the Faculty of Medicine Siriraj Hospital. Peripheral blood mononuclear cells (PBMCs) were isolated by density gradient centrifugation in Lymphocyte Separation Medium (Corning, Inc., New York, NY, USA). Monocyte-depleted PBMCs were cultured in TexMACS™ Medium (Miltenyi Biotec, Bergisch Gladbach, Germany) supplemented with 5% human AB serum (Sigma-Aldrich) and 100 U/mL of recombinant human interleukin (rhIL)-2, 5 ng/ml of rhIL-7, and 20 ng/ml of rhIL-15 (Immunotools, Friesoythe, Germany). T cells were activated with 5 μg/ml Phytohemagglutinin-L (PHA-L) (Roche, Basel, Switzerland) for 72 h. The activated T cells were transduced with lentiviruses-containing a scFv-CAR in the presence of 10 μg/mL of protamine sulfate (Sigma-Aldrich) and spinoculated at 1,200 g for 90 min at 32°C. The transduction efficiency of CAR^+^ cells was determined by fluorescein isothiocyanate (FITC)-labeled anti-Myc antibody staining and flow cytometry analysis. The expression of CAR protein in T cells was examined by immunoblot analysis (see details in **Supplementary Material**).

### Target Cell Killing Assay

The killing of HeLa-CD19^+^ cells was evaluated by a crystal violet staining assay. HeLa-CD19^+^ cells were co-cultured with CAR T cells at multiple E:T ratios for 24 h. After the effector cells were removed, crystal violet staining was performed. The plate was washed and the cell-bound dye was dissolved in 1% sodium dodecyl sulfate (SDS) solution. Absorbance was measured at a wavelength of 570 nm using a Sunrise™ Absorbance Microplate Reader (Tecan Group Ltd., Männedorf, Switzerland), and the data were analyzed using Magellan™ data analysis software version 6.6.0.1 (Tecan).

To evaluate cytotoxic effect of anti-CD19-CAR T cells on Raji cells, a flow-based assay using counting beads (123count™ eBeads Counting Beads; Thermo Fisher Scientific, CA, USA) was performed to numerate the remaining tumor cells in the culture. In brief, transduced CAR-T cells were co-cultured with Raji cells that were labeled with 1 µM CellTracker™ CMFDA Dye at an E:T ratio of 1:1. After 24 and 48 hours of co-culturing, the absolute number of tumor cells were counted by flow cytometry using the counting beads as control. Absolute cell number was calculated by the following formula: (cell count x eBead volume/eBead count x cell volume) x eBead concentration. The following equation was used to calculate the percentage of cytotoxic activity: [1-(target cell in each condition/target cell alone at the indicated time)] x 100.

The killing of anti-CD19-CAR T cells against CD19^+^ target cells were determined by using a flow cytometry-based cytotoxicity assay using Raji and K562-CD19^+^ cell lines overexpressing CD19 as target cells, and using K562-CD19^-^ cell line as a negative control. These target cells were labeled with carboxyfluorescein succinimidyl ester (CFSE; Thermo Fisher Scientific) and co-cultured with CAR T cells at various effector to target (E:T) ratios for 4 h. After co-culturing, the cells were collected and stained with annexin V-APC and propidium iodide (PI) (Immunotools). The condition of target cells culturing alone was determined as spontaneous target cell death. The killing efficacy of CAR T cells was calculated using the following equation: % specific killing = [(experimental target cell death-spontaneous target cell death)/(100-spontaneous target cell death)]x100.

### Proliferation Assay

CAR-transduced T cells (CAR T) and mock-transduced T cells (Mock T) were labeled with 0.5 μM CFSE (Thermo Fisher Scientific) and co-cultured with Raji cells at an E:T ratio of 5:1 in the absence of exogenous cytokines. After 72 h of co-cultivation, effector T cell proliferation was examined by flow cytometry by gating the lymphocyte population to evaluate CFSE dilution as the result of cell proliferation. The collected data were analyzed by the software installed in the machine, and by FlowJo 10 software (FlowJo, RRID : SCR_008520).

### Cytokine Production

Cytokines, including IFN-γ, TNF-α and IL-2, that were secreted from CAR T cells in response to exposure to CD19^+^ target cells were determined by enzyme-linked immunosorbent assay (ELISA). CAR T cells or Mock T cells (2x10^5^) were co-cultured with Raji cells (4x10^4^) in a complete medium without the addition of exogenous cytokines. After co-culturing for 24 h, IFN-γ, TNF-α and IL-2 levels in the culture supernatant were determined by Quantikine^®^ ELISA (R&D System, Inc., Minneapolis, MN, USA).

### Statistical Analysis

Datasets from at least 3 independent experiments were gathered, and the results are expressed as mean ± standard error of the mean (SEM). GraphPad Prism 7.0 (GraphPad Prism, RRID : SCR_002798) was used for all statistical analyses. Student’s *t*-test and one-way analysis of variance (ANOVA) was used to compare two groups of an experimental study, and more than two groups of an experimental study, respectively. A *p*-value less than 0.05 was considered to be statistically significant.

## Results

### Generation and Characterization of mFMC63-CAR2 T Cells, mFMC63-CAR3 T Cells and mFMC63-CAR4 T Cells Targeting CD19

**Figure 1:**
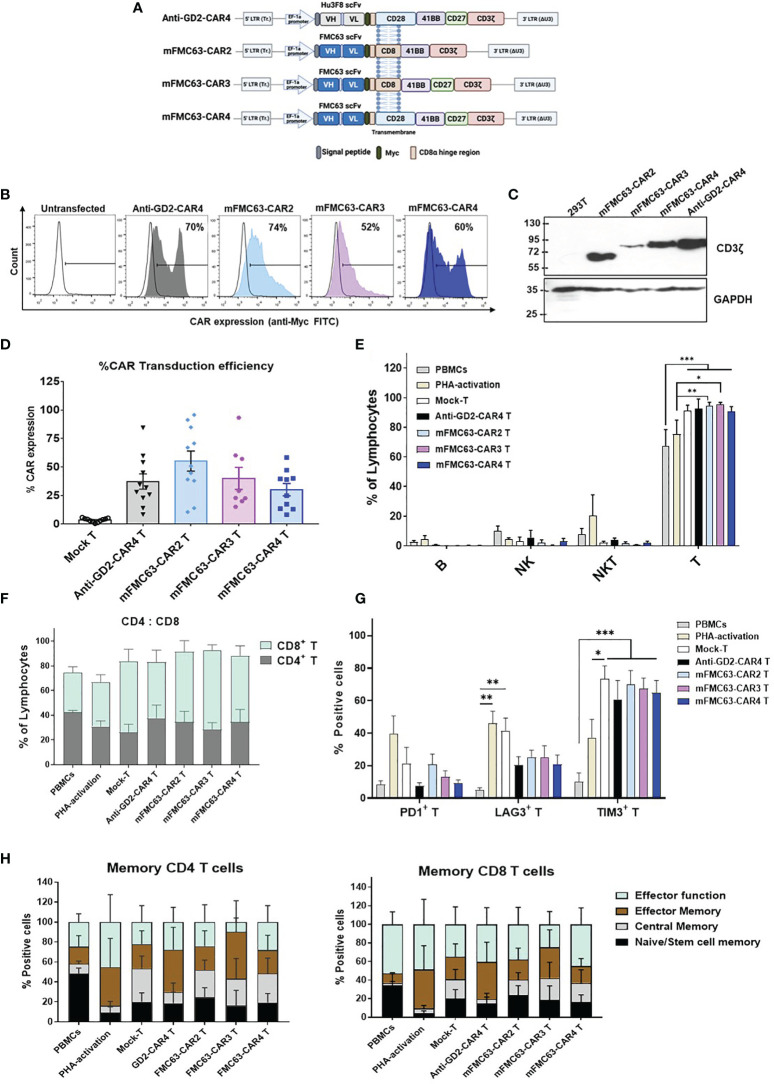
Schematic representation of CAR constructs, CAR protein expression, and immunophenotypes of CAR T cells. **(A)** Schematic representation of the second-generation (CAR2), third-generation (CAR3), and fourth-generation CAR (CAR4) lentiviral constructs that contain a signal peptide (SP), antigen binding domain (scFv) derived from murine monoclonal antibody–mFMC63 (middle and bottom) or Hu3F8 of anti-GD2 (top), Myc epitope tag, hinge region, transmembrane (TM) domain, costimulatory domain(s), and CD3ζ. **(B)** Histogram of CAR expression on cell surface of transfected HEK293T cells examined by flow cytometry method using anti-Myc (fluorescein isothiocyanate) FITC staining. **(C)** Immunoblot analysis of CAR protein expression in HEK293T cell lysates. **(D)** Percentages of CAR expression on the T cell surface (n=11). **(E)** Immune cell populations in the experiments, including B, NK, NKT, and T cells. **(F)** Percentages of helper (CD4^+^) T cells and cytotoxic (CD8^+^) T cells. **(G)** Exhaustion marker profiles of CAR-transduced T cells. **(H)** Subpopulations of CD4^+^ T cells and CD8^+^ T cells of CAR T cells. Characterization of CAR T cells was conducted after lentiviral transduction for 5 days. Data are shown as the mean ± standard error of the mean (SEM) of at least 3 independent experiments using blood samples from at least three healthy volunteer donors. One-way analysis of variance (ANOVA) was used to determine statistical significance (*p < 0.05, **p < 0.01, ***p < 0.001). The images of the lentiviral constructs were created using the web-based program BioRender.com.

The reported poor clinical outcomes due to decreased efficacy of anti-CD19-CAR2 T cells in clinical trials influenced our endeavor to improve anti-CD19-CAR T functions. We, therefore, generated the mFMC63-CAR4 (28BB27ζ). The mFMC63-CAR2 (BBζ) and mFMC63-CAR3 (BB27ζ) were also generated for comparison, and the Hu3F8-CAR4 or anti-GD2-CAR4 was generated as an irrelevant CAR4 control (**Figure 1A**). The expression of CAR protein on HEK293T cell surface (**Figure 1B** and **Supplementary Figure 1**) and in cell lysates were demonstrated (**Figure 1C**). The transduction efficiencies in lymphocytes were determined by CAR surface expression. The percentages of CAR-expressing lymphocytes were 37.16 ± 6.67% for anti-GD2-CAR4, 55.14 ± 8.83% for mFMC63-CAR2, 40 ± 9.69% for mFMC63-CAR3, and 30.1 ± 5.36% for mFMC63-CAR4 (**Figure 1D**). Immunophenotypes of CAR T cells were characterized (**Supplementary Figure 3**). The populations of T cells were more than 90% after the lentiviral transduction process (**Figure 1E**). Moreover, in all lentiviral-transduced conditions, the proportion of cytotoxic (CD8^+^) T cells to helper (CD4^+^) T cells was approximately 75:25 compared with 33:67 in PBMC, and 40:60 in PHA activation (**Figure 1F**). In addition, PHA-activated T and Mock-T cells exhibited higher levels of LAG3 compared to the PBMCs (**Figure 1G**). However, the expression of TIM3 was increased in Mock-T cells (73.24 ± 8.21%, *p*<0.0001), mFMC63-CAR2 T cells (69.79 ± 8.75%, *p*<0.0001), mFMC63-CAR3 T cells (67.37 ± 6.65%, *p*=0.0002), and mFMC63-CAR4 T cells (65.00 ± 7.27%, *p*<0.0001), compared to PBMCs (10.00 ± 5.60%) (**Figure 1G**). The memory phenotypes of CD4^+^ T cell and CD8^+^ T cell were identified by expression of CD45RO and CD62L which were increased in both CD4^+^ T cells and CD8^+^ T cells of CAR T cell products (**Figure 1H** and **Supplementary Figure 4**).

### mFMC63-CAR4 T Cells Exhibited Higher Anti-Tumor Efficiency Than mFMC63-CAR2 and mFMC63-CAR3 T Cells

**Figure 2:**
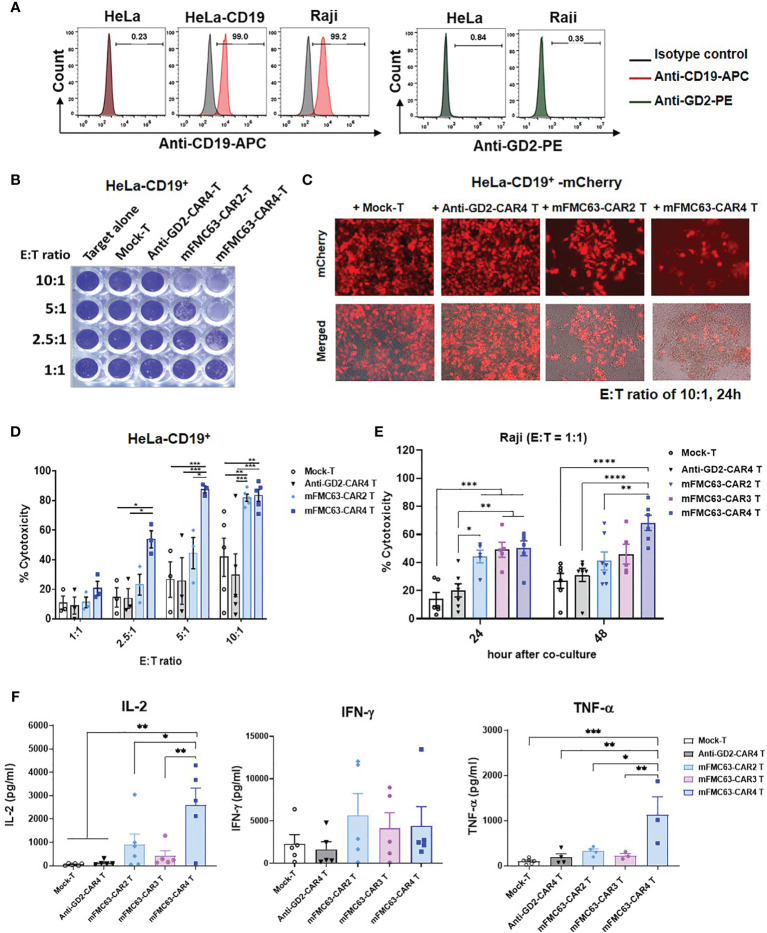
Anti-tumor activities of mFMC63-CAR T cells. **(A)** Histograms of flow cytometry showed CD19 antigen expression on Raji cells, parental HeLa cells, and engineered HeLa-CD19^+^ cells; however, the GD2 antigen was absent on these cells. **(B)** Crystal violet staining of the remaining HeLa-CD19^+^ target cells after co-culturing with Mock-T cells, anti-GD2-CAR4 T cells, mFMC63-CAR2 T cells, or mFMC63-CAR4 T cells at effector to target (E:T) ratios of 1:1, 2.5:1, 5:1, and 10:1 for 24 hours (h). **(C)** Fluorescence images of HeLa-CD19^+^ expressing mCherry co-cultured with CAR T cells at an E:T ratio of 10:1 as analyzed by fluorescence microscopy. **(D)** Bar graphs represent the percentages of cytotoxicity against HeLa-CD19^+^ target cells by CAR T cells as measured by crystal violet staining assay. **(E)** Cytotoxicity of Raji cells expressing CD19 antigen by CAR T cells after co-culturing at an E:T ratio of 1:1 for 24 and 48 h. **(F)** Levels of secreted IL-2, IFN-γ, and TNF-α cytokines in the culture supernatants of co-cultured CAR T cells and Raji cells. Data are presented as the mean ± standard error of the mean (SEM) from at least three independent experiments using blood samples from three healthy volunteer donors. One-way analysis of variance (ANOVA) was used to determine statistical significance (*p < 0.05, **p< 0.01, ***p < 0.001, ****p < 0.0001).

To compare the anti-tumor efficiencies of mFMC63-CAR2 T cells, mFMC63-CAR3 T cells, and mFMC63-CAR4 T cells, an *in vitro* killing assay against CD19^+^ target cells was conducted. Both Raji and HeLa-CD19^+^ cells expressed CD19 on their cell surface. There was no GD2 expression in any target cell tested (**Figure 2A**). HeLa-CD19^+^ cells were co-cultured with CAR T cells at various E:T ratios for 24 h. After co-culturing, the viable target cells attached to the culture plate were stained with crystal violet dye (**Figure 2B**). HeLa-CD19^+^ cells were engineered to express a red fluorescent protein, mCherry, and used to evaluate the killing efficiency under fluorescence microscopy. The result of co-culturing at an E:T ratio of 10:1 for 24 h demonstrated that mFMC63-CAR2 T cells could kill mCherry-HeLa-CD19^+^ cells less than that of mFMC63-CAR4 T cells (**Figure 2C**). The cytotoxicities of mFMC63-CAR2 T cells and mFMC63-CAR4 T cells were increased in a dose-dependent manner. The mFMC63-CAR2 T cells could lyse HeLa-CD19^+^ cells less than that of mFMC63-CAR4 T cells at an E:T of 1:1 to 5:1 (**Figure 2D**).

To further compare the cytotoxicities of mFMC63-CAR2, mFMC63-CAR3, and mFMC63-CAR4 T cells, Raji cells that natively expressed CD19 antigen on the cell surface were used. The killing of Raji (CD19^+^) by CAR T cells was evaluated by flow cytometry using counting bead assay. Raji (CD19^+^) cells were labeled with fluorescent dye before co-cultivating with CAR T cells at an E:T ratio of 1:1 for 24 h and 48 h. After co-culturing for 24 h, the results showed significantly increased cytotoxicities of mFMC63-CAR2 T cells (44.25 ± 4.52%, *p*=0.0038), mFMC63-CAR3 T cells (49.08 ± 5.42%, *p*=0.0006), and mFMC63-CAR4 T cells (50.16 ± 5.33%, *p*=0.0002) than those killed by Mock-T (13.87 ± 4.73%) (**Figure 2E**). Moreover, the killing of mFMC63-CAR3 T cells (*p*=0.0046) and mFMC63-CAR4 Tcells (*p*=0.0016) were significantly higher than those killed by anti-GD2-CAR4 T cells (20.22 ± 4.74%). Interestingly, after the co-culturing for 48 h, the killing of Raji cells by mFMC63-CAR4 T cells (68.23 ± 5.53%) was higher than those killed by mFMC63-CAR2 T cells (41.15 ± 6.44%, *p*=0.0053), or anti-GD2-CAR4 cells (31.07 ± 4.65%, *p*<0.0001), or Mock-T cells (26.93 ± 5.30%, *p*<0.0001) (**Figure 2E**). Although the killing of Raji cells by mFMC63-CAR3 T cells was not significantly different from that of mFMC63-CAR4 T cells, the killing efficiency of mFMC63-CAR3 T cells seemed to be lower than that of mFMC63-CAR4 T cells (**Figure 2E**).

To examine cytokine secretion by CAR T cells, Raji (CD19^+^) cells were co-cultured with CAR-T cells at an E:T ratio of 5:1 for 24 h. After the co-culturing, the levels of IL-2 secreted by Mock-T cells, anti-GD2-CAR4 T cells, mFMC63-CAR2 T cells, mFMC63-CAR3 T cells, and mFMC63-CAR4 T cells were 59.18 ± 17.87 pg/ml, 133.0 ± 46.70 pg/ml, 903.1 ± 456.3 pg/ml, 425.7 ± 217.6 pg/ml, and 2,599 ± 726.10 pg/ml (*p*=0.0093), respectively. The level of IFN-γ secreted by Mock-T cells, anti-GD2-CAR4 T cells, mFMC63-CAR2 T cells, mFMC63-CAR3 T cells, and mFMC63-CAR4 T cells were 2,304 ± 1078 pg/ml, 1,645 ± 895.9 pg/ml, 5,668 ± 2556 pg/ml, 4,149 ± 1810 pg/ml and 4,438 ± 2265 pg/ml, respectively (**Figure 2F**). The level of TNF-α secreted by mFMC63-CAR4 T cells showed significantly higher than those secreted from other CAR T cells (*p ≤* 0.01). However, we did not observe the difference of TNF-α levels secreted by mFMC63-CAR2 and mFMC63-CAR3 T cells (**Figure 2F**).

### Identification of Human scFv Specifically Binding to CD19 by Screening From Human scFv Bacteriophage Display Library

**Figure 3:**
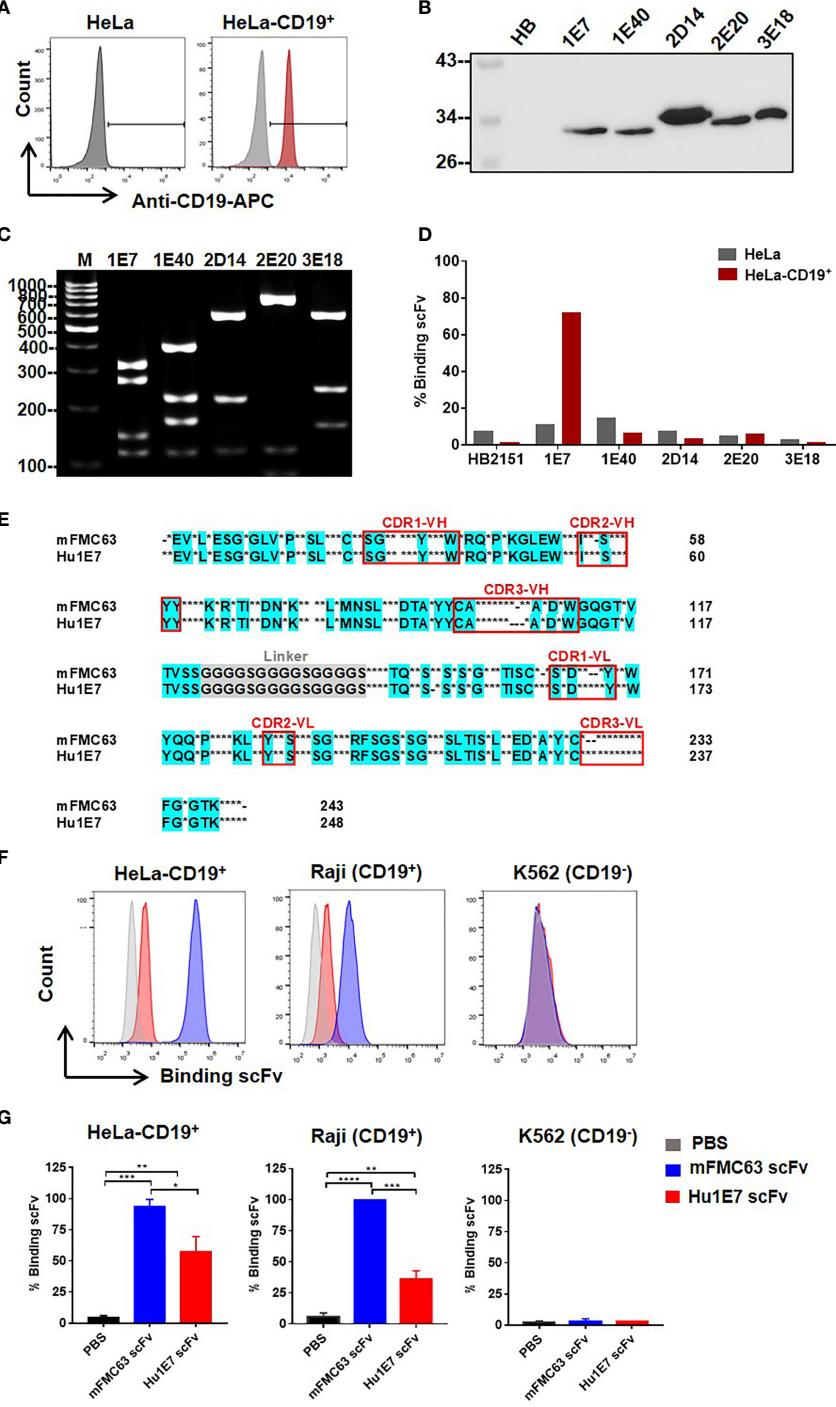
Identification of novel human scFv specifically binding to CD19 antigen from a human scFv bacteriophage display library. **(A)** CD19 antigen expression on HeLa-CD19^+^ cell surface. **(B)** Detection of the selected human scFv proteins expressed and secreted in bacterial culture supernatants by immunoblot analysis using monoclonal anti-Myc antibody. **(C)** Digested DNA patterns of scFv clones analyzed on agarose gel. **(D)** Evaluation of human scFv binding to HeLa cells (gray-colored bar) and HeLa-CD19^+^ cells (red-colored bar) by flow cytometry. **(E)** Amino acid sequence of Hu1E7 scFv selected from a human scFv bacteriophage display library by biopanning against HeLa-CD19^+^. The coding sequence of scFv was analyzed using an immunoglobulin variable domain sequence analysis tool (IgBLAST) (https://www.ncbi.nlm.nih.gov/igblast/). The amino acid sequence of Hu1E7 scFv was aligned with that of murine-derived scFv (mFMC63). Conserved sequences in the alignments are in cyan color. Dots indicate different amino acids, and dashes indicate amino acid deletion. The complementarity determining regions (CDRs) of the variable domains are indicated in the red box. **(F)** Histogram and **(G)** Bar graphs of the binding abilities of the recombinant mFMC63 scFv (blue) and Hu1E7 scFv (red) to CD19 antigen expressed on the cell surface of HeLa-CD19^+^, Raji and K562 cells as analyzed by flow cytometry. Data are presented as the mean ± standard error of the mean (SEM) from at least three independent experiments. One-way analysis of variance (ANOVA) was used to determine statistical significance (*p < 0.05, **p < 0.01, ***p < 0.001, ****p < 0.0001).

To identify human scFv specifically binding to the CD19, a human scFv bacteriophage display library (Yamo-I) (28) was used for biopanning against CD19 antigen expressed on HeLa-CD19^+^ cells. Initially, HeLa cells, tightly adherent cells on the plating vessel, were generated to stably express the human CD19, as illustrated in **Figure 3A**. The output of the screening is shown in **Supplementary Table 1**. Finally, 5 out of 288 phage clones, designated as 1E7, 1E40, 2D14, 2E20, and 3E18, were found to express soluble scFv proteins in the bacterial culture media (**Figure 3B**). These 5 scFv clones displayed 5 different *Mva*I digested-DNA patterns (**Figure 3C**). Next, these 5 scFv proteins were examined for their binding ability to CD19 on HeLa-CD19^+^ cells by flow cytometry. The result demonstrated that only the 1E7 scFv clone showed good binding to the CD19 on HeLa-CD19^+^ cells (72%, MFI=585), when compared to the parental HeLa cells (11.13%, MFI=100). The other 4 scFv clones could not bind to either cell line (**Figure 3D**).

The immunoglobulin gene sequence of *Hu1E7 scFv* was validated by IMGT software (http://www.imgt.org/IMGT_vquest/vquest). The *Hu1E7 scFv* gene contained 744 nucleotides and was predicted to encode 248 amino acids. The deduced amino acids of *Hu1E7 scFv* included variable heavy chain (VH), (G4S)x3-linker, and variable light chain (VL). *Hu1E7 scFv* contained a complete sequence of both VH and VL sequences comprising 4 framework regions (FR) and 3 complementarity-determining regions (CDR). VH of Hu1E7 shares an identical VH sequence with the germline sequence IGHV3-21*01 (NCBI accession no. NC_000014.9, IMGT accession no. AB019439). Analysis of the VL sequence showed that Hu1E7 VL was a lambda chain which shares 95.5% identity with the germline sequence IGLV2-14*01 (**Supplementary Table 2**). The amino acid sequences alignment of mFMC63 scFv and Hu1E7 scFv was shown in **Figure 3E** and **Supplementary Table 3**.

To compare the binding abilities of the mFMC63 scFv and Hu1E7 scFv, these two proteins were overexpressed in CHO-K1 cells. The purified Hu1E7 scFv and mFMC63 scFv were incubated with HeLa-CD19^+^, Raji (CD19^+^), and K562 (**Figure 3F**, **Supplementary Figure 4**). The mFMC63 scFv bound to 100% of HeLa-CD19^+^ and Raji (CD19^+^). The Hu1E7 scFv bound 69% and 44% of HeLa-CD19^+^ and Raji (CD19^+^), respectively (**Supplementary Figure 4**). Specific binding of the mFMC63 scFv and Hu1E7 scFv to the CD19 was demonstrated by their relative inability to bind to K562 cells (**Figures 3F**, **G**).

### Generation and Characterization of mFMC63-CAR4 T Cells and Hu1E7-CAR4 T Cells

**Figure 4:**
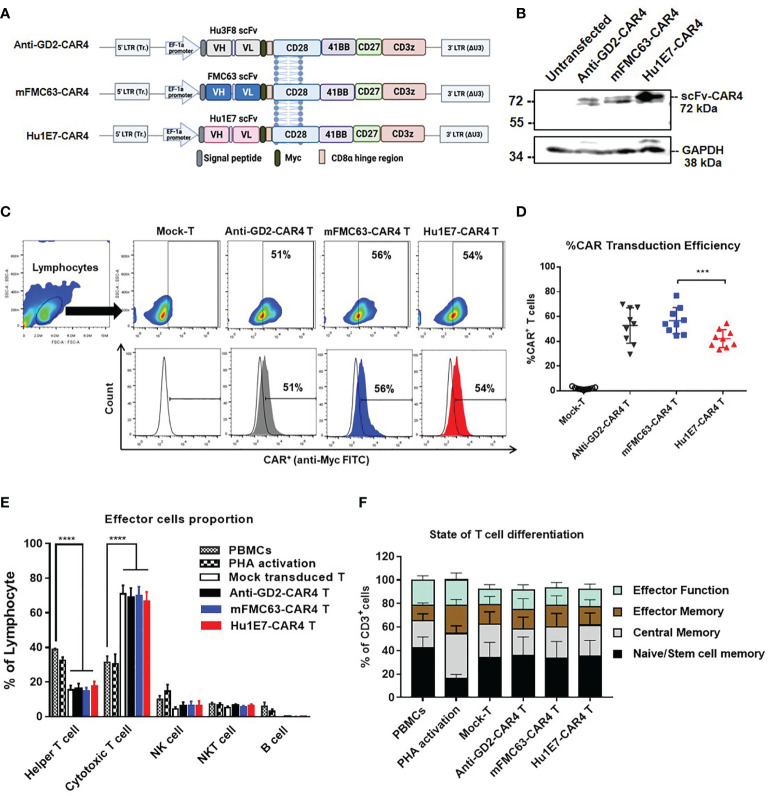
Schematic diagrams of CAR4 constructs in lentiviral vector, CAR4 protein expression, immunophenotypes, and subpopulations of CAR4 T cells. **(A)** Schematic representations of anti-GD2-CAR4, mFMC63-CAR4, and Hu1E7-CAR4 constructs containing a signal peptide (SP), scFv, Myc epitope tag, CD8α hinge region, CD28 as transmembrane (TM) domain, and costimulatory signaling domains, including CD28, 4‐1BB, CD27, and CD3ζ. **(B)** Immunoblot analysis of CAR4 proteins expressed in HEK293T cells. **(C)** Pseudocolor plot and histograms from flow cytometry are represented the gating strategy of surface CAR protein expression on transduced T cells. **(D)** Summary of dot plots showing the transduction efficiency of CAR constructs to express CAR protein on T cells isolated from peripheral blood mononuclear cells (PBMCs) from healthy volunteer donors (n=9). **(E)** Immunophenotypes of lymphocyte populations. **(F)** States of T cell differentiation. The data were obtained from at least 3 independent experiments, and the results are shown as mean ± standard error of the mean (SEM). One-way analysis of variance (ANOVA) was used to determine statistical significance (***p < 0.001, ****p < 0.001).

Lentiviral constructs were generated to contain *Hu1E7 scFv* sequences fused upstream to the CAR4 cassette genes and encoding a Myc-tagged peptide, the human CD8α hinge region, the CD28 transmembrane region, and intracellular domains of CD28, 4-1BB, CD27, and CD3ζ (**Figure 4A**). The anti-GD2-CAR4 was used as a control in the following experiments. Protein expression of CAR4 construct was examined by an immunoblotting technique using an anti-CD3ζ antibody. These three CAR4 proteins could be expressed with an expected size of 72 kDa, where GAPDH was used as a loading control (**Figure 4B**). Approximately 40-50% of the T cells expressed anti-GD2-CAR4, mFMC63-CAR4, or Hu1E7-CAR4 on their cell surface (**Figure 4C, D**). The transduction efficiency of Hu1E7-CAR4 T cells (42.16 ± 2.4%) was significantly lower than that of mFMC63-CAR4 T cells (56.61 ± 3.53%, *p*=0.0004) (**Figure 4D**). The immunophenotype of CAR T cells showed a significantly increased proportion of CD8^+^ T cells (*p*<0.0001) while the CD4^+^ T cells were significantly decreased (*p*<0.0001) compared to PBMC and activated T cells (**Figure 4E**). The memory T cells phenotype identified by the expression of CD45RO and CD62L (**Supplementary Figure 5**), were similar to those of PBMC (**Figure 4F**). These results indicated that anti-CD19-CAR4 T cells were successfully generated, and memory phenotypes of T cells were still maintained to be similar to those of PBMC.

### Killing Activities of mFMC63-CAR4 T Cells and Hu1E7-CAR4 T Cells Against Cancer Cell Lines Expressing CD19

**Figure 5:**
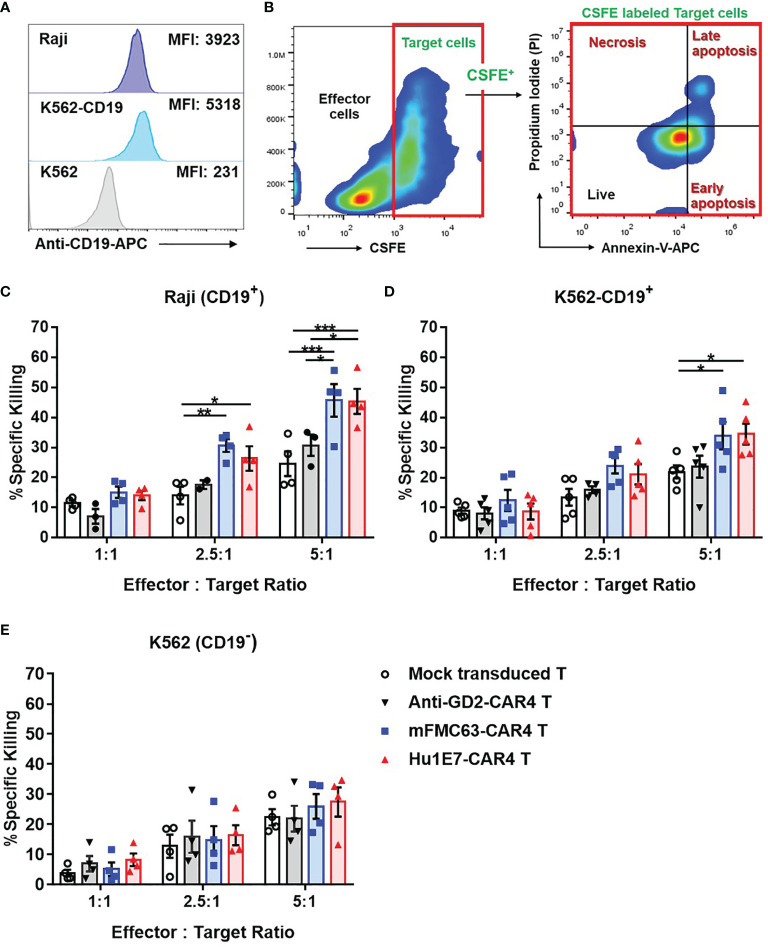
The killing activities of mFMC63-CAR4 T cells and Hu1E7-CAR4 T cells against three target cancer cell lines, including Raji (CD19^+^), K562-CD19^+^, and K562 (CD19^-^) cell lines. **(A)** CD19 antigen expression on Raji (CD19^+^), K562-CD19^+^, and K562 (CD19^-^) cells. **(B)** Gating strategy and annexin V/PI staining to analyze target cancer cell death used in cell killing assay. The killing activities of Mock-T cells, anti-GD2-CAR4 T cells, mFMC63-CAR4 T cells, and Hu1E7-CAR4 T cells against **(C)** Raji (CD19^+^), **(D)** K562-CD19^+^, and **(E)** K562 (CD19^-^) cells after co-culturing at effector to target (E:T) ratios of 1:1, 2.5:1, and 5:1 for 4 hours. Data from at least 3 independent experiments were shown as mean ± SEM. The statistically significant levels are: The data were obtained from at least 3 independent experiments, and the results are shown as mean ± standard error of the mean (SEM). One-way analysis of variance (ANOVA) was used to determine statistical significance (*p < 0.05, **p < 0.01, ***p < 0.001).

The killing activities of anti-CD19-CAR4 T cells against three cancer cell lines were examined (**Figure 5A**). Target cell death was defined as shown in **Figure 5B**. Regarding the killing of Raji (CD19^+^) cells, the killing activities of mFMC63-CAR4 T cells and Hu1E7-CAR4 T cells were comparable. The specific killing at an E:T ratio of 5:1 of both mFMC63-CAR4 T cells (45.74 ± 5.41%) and Hu1E7-CAR4 T cells (45.42 ± 4.18%) were significantly higher than that of Mock-T cells (*p*=0.0003 and 0.0004, respectively) (**Figure 5C**). At an E:T ratio of 2.5:1 of both mFMC63-CAR4 T cells (30.65 ± 2.11%) and Hu1E7-CAR4 T cells (26.34 ± 4.12%) were also significantly higher than that of Mock-T cells (*p*=0.0046 and 0.0485, respectively) (**Figure 5C**). The K562-CD19^+^ killing activities of mFMC63-CAR4 T cells and Hu1E7-CAR4 T cells at an E:T ratio of 5:1 were also similar (34.03 ± 4.54% vs 34.54 ± 3.46%), which were both higher than that of Mock-T cells (*p*=0.0188 and *p*=0.0259, respectively) (**Figure 5D**). Regarding the killing of K562 (CD19^-^), the percentages of specific killing increased commensurate with increases in the E:T ratio. However, no statistically significant killing activities were observed compared to those of the control Mock-T cells or anti-GD2-CAR4 T cells (**Figure 5E**).

### Cell Proliferation and Cytokine Production of mFMC63-CAR4 T Cells and Hu1E7-CAR4 T Cells

**Figure 6:**
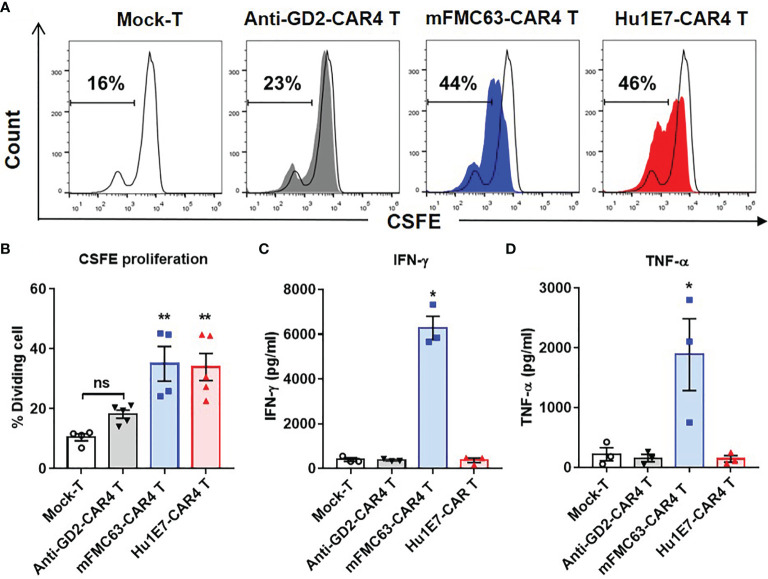
Cell proliferation and cytokine production of anti-CD19 CAR T cells. **(A)** Cell proliferation of Mock-T cells, anti-GD2-CAR4 T cells, mFMC63-CAR4 T cells, and Hu1E7-CAR4 T cells after activation by co-culturing with Raji (CD19^+^) cells at an effector to target (E:T) ratio of 5:1 for 72 hours (h) in the absence of exogenous cytokines were examined by carboxifluorescein diacetate succinimidyl ester (CFSE) dilution and flow cytometry. **(B)** Histogram showing the percentages of cell proliferation of Mock-T cells, anti-GD2-CAR4 T cells, mFMC63-CAR4 T cells, and Hu1E7-CAR4 T cells. The data were obtained from at least 3 independent experiments using T cells from 3 healthy volunteer donors, and the results are shown as mean ± standard error of the mean (SEM). The data of Mock-T cells were compared with those of others. Statistically significant differences were determined by one-way analysis of variance (ANOVA) (**p < 0.01). **(C)** The levels of IFN-γ and **(D)** TNF-α production in the cell culture supernatants of anti-CD19 CAR T cells after activation by culturing with Raji (CD19^+^) cells at an E:T ratio of 5:1 for 24 h were analyzed by enzyme-linked immunosorbent assay (ELISA). The data were obtained from at least 3 independent experiments using T cells from 3 healthy volunteer donors. The data of Mock-T cells were compared with those of others. One-way ANOVA was used to analyze for statistically significant differences (*p < 0.05). ns, not significant.

To investigate T cell proliferation, CAR T cells were labeled with CFSE and co-culturing with Raji (CD19^+^) cells. The CFSE dilution profile was analyzed by flow cytometry. After co-culturing, mFMC63-CAR4 T cells and Hu1E7-CAR4 T cells showed 44% and 46% proportion of cell proliferation, respectively (**Figure 6A**). In the study including three subjects, similar proportions of significantly different cell proliferation between mFMC63-CAR4 T cells (35 ± 5.76%) and Hu1E7-CAR4 T cells (33.94 ± 4.53%) were observed when compared to Mock-T cells (*p*=0.002 and *p*=0.003, respectively). It should be noted that anti-GD2-CAR4 T cells showed no CD19-dependent proliferation compared to that of Mock-T cells (**Figure 6B**). To examine cytokine (IFN-γ and TNF-α) secretion from CAR T cells after co-culturing with Raji (CD19^+^) cells, the culture supernatants were collected and quantified by ELISA. The results showed that, compared to Mock-T cells, mFMC63-CAR4 T cells secreted a significantly higher level of IFN-γ (6,277 ± 524.8 pg/ml, *p*=0.0237) and TNF-α (1,888 ± 601.3 pg/ml, *p*=0.0301). The levels of IFN-γ and TNF-α secreted by Hu1E7-CAR4 T cells were 327.7 ± 103.8 pg/ml and 144.5 ± 55.2 pg/ml, respectively (**Figures 6C, D**).

## Discussion

Currently, there are four anti-CD19-CAR T cell products that have been approved for clinical use (2, 6–8). These products are second-generation CAR (CAR2) containing an anti-CD19 scFv derived from murine (FMC63) (11). The short *in vivo* persistence of anti-CD19-CAR2 T cells is a crucial factor of CD19^+^ relapses (2, 30, 31). The different CAR design critically determined the efficiency of CAR T cells and their anti-tumor functions (32–34). Comparison among CAR2 T cells, CD28-based CAR exhibited rapid expansion and strong effector-like function, but shorter T cell persistence (35) whereas 4-1BB-based CAR had a superior function in promoting more memory phenotype, which prolonged anti-tumor activity of CAR T cells *in vivo* (14, 36). Moreover, CD27-based CAR T cells promoted long-term anti-tumor activity *in vivo* by upregulation of the anti-apoptotic protein Bcl-XL (16). We hypothesized that the limitation of the CAR2 containing only one costimulatory domain could be overcome by the creation of a fourth-generation CAR (CAR4) that included three costimulatory domains. This new CAR4 construct has been reported to possess a potent anti-tumor efficiency against some solid tumor models, for examples, anti-FRα-CAR4 T cells in breast cancer (21), anti-MUC1-CAR4 T, anti-CD133-CAR4 T, and A20-CAR4 T cells in cholangiocarcinoma cells (18–20).

To test this hypothesis, we created mFMC63-CAR2 (BBζ) T cells, mFMC63-CAR3 (BB27ζ), and mFMC63-CAR4 (28BB27ζ) T cells (**Figure 1**). In the CAR T cell products, the main population (90%) were T cells with a CD8:CD4 ratio of 3:1. The change in CD8:CD4 ratio from the original PBMC might occur from induction by PHA-L and IL-2 supplementation in the culture medium (37), which favorably supports CD8 T cell proliferation. The memory T cells were increased in both CD4^+^ and CD8^+^ subpopulations of the CAR T cell products, which have excellent proliferation and *in vivo* functional activities (30). When the anti-tumor efficiencies against HeLa-CD19^+^ cells were compared, mFMC63-CAR2 T cells elicited the lower cytolytic activity than that of mFMC63-CAR4 T cells. Furthermore, the long-term killing activity against Raji (CD19^+^) cells was evaluated. mFMC63-CAR4 T cells displayed markedly higher cytolytic activity than that of mFMC63-CAR3 T cells and mFMC63-CAR2 T cells at 24 and 48 h of co-cultivation (**Figure 2E**). The CD19-specific TH1 cytokine produced by CAR T cells was measured. The results showed that mFMC63-CAR4 T cells secreted more IL-2 and TNF-α cytokines than those secreted by mFMC63-CAR2 or mFMC63-CAR3 T cells. These data indicated the anti-tumor efficiency of anti-CD19-CAR4 T cells, which is consistent with the results of our previous studies (18–20).

To reduce the immunogenicity of the CAR construct, the anti-CD19-CAR2 T cells expressing humanized scFv (hCD19-CAR T cells) were investigated (23). However, some murine CDR sequences in humanized scFv could induce an immune response to CAR T cells causing tumor relapse (27). Thus, anti-CD19-CAR containing fully human scFv would be more efficient for clinical use. The fully human anti-CD19-CAR2 T cells carrying either CD28 (38) or 4-1BB (39) were previously reported. Sommermeyer and colleagues also identified a human scFv-CD19 from a human antibody chain library derived from the bone marrow and PBMCs of twenty donors (39). That work, however, focused only on scFv that shared epitopes with FMC63 (39). There was evidence of tumor escape caused by CD19 mutation (40, 41); thus, FMC63-CAR T cells were unable to recognize the mutant CD19. A search for human scFv-CD19 that binds to different epitopes of the CD19 for CAR T cell production would overcome the problem of immunogenicity and resistance associated with the use of mFMC63 scFv.

To screen for a novel human scFv, we generated HeLa-CD19^+^ that could be used as a source of near-native CD19 for the screening. The human scFv phage display (Yamo-I) library that was previously generated by Pansri et al. (28) was used. This human scFv library is a compact library, prepared from rearranged V-gene, and assembled *via* the use of gene repertoires from 140 healthy donors. Yamo-I library might provide antigen-specific human scFv that are relatively close to human antibody germline with less extended somatic hypermutation (42). After biopanning, a fully human scFv-CD19 (Hu1E7) showed specific binding to CD19 expressed on HeLa-CD19^+^ cells, but it did not bind to the parental HeLa cells (**Figure 3D**). The results of comparison between the *Hu1E7 scFv* sequence and the closest germline V sequence of known human antibodies showed that *Hu1E7 scFv* shares an identical VH sequence with the germline sequence of IGHV3-21*01. There was no nucleic acid sequence of *Hu1E7 VH* different from the germline sequence known to be immunogenic in humans (42). Analysis of the VL sequence showed that Hu1E7 contains VL from the lambda chain, which shares 95.50% with the germline sequence of IGLV2-14*01 with 13 amino acids different from the germline protein. The number of different amino acids of the VL from the germline protein suggests that the VH of Hu1E7, which was derived from the germline B gene, assembled to a VL sequence derived from the antibody of secondary immune response, generating a novel scFv that could bind to CD19, which is a self-antigen. The binding abilities of recombinant mFMC63 scFv and Hu1E7 scFv were evaluated (**Figure 3F**). The Hu1E7 scFv showed lower binding abilities to HeLa-CD19^+^ cells and Raji (CD19^+^) cells, but no binding to K562, which indicates its specificity to the CD19 (**Figures 3F–G**). Further studies are needed to map binding epitopes on CD19 of these two scFvs, and to identify the distinct roles of amino acids within the CDRs. At this point, we were able to identify a fully human scFv (Hu1E7) that is specific to the CD19, which yields the advantage of being an entirely human sequence with a lower risk of transgene immunogenicity.

Lentiviral constructs of Hu1E7 scFv were generated to contain the CAR4 (28BB27ζ) in the same VH-VL orientation to mFMC63 scFv (**Figure 4**). Both mFMC63-CAR4 T cells and Hu1E7-CAR4 T cells expressed comparable CAR proteins on the T cell surface. All CAR4 T cells contained a similar memory T cell phenotype to those of PBMCs. These results indicated that Hu1E7-CAR4 T cells were successfully generated, and they presented similar characteristics of T cells to those of mFMC63-CAR4 T cells. The killing of Hu1E7-CAR4 T cells against Raji (CD19^+^) cells was comparable to the killing of mFMC63-CAR4 T cells. Similar results were also found in the co-cultivation with K562-CD19^+^ cells. The cytolytic activities against K562-CD19^-^ cells were not statistically significantly different when compared with Mock-T and anti-GD2-CAR4 T cells, which indicates the specificity of anti-CD19-CAR4 T cells (**Figure 5**). Our results demonstrated that Hu1E7-CAR4 T cells had comparable cytolytic activity and specificity to those of mFMC63-CAR4 T cells even though the recombinant Hu1E7 scFv showed lower binding ability to the CD19.

The efficacy of CAR T cell therapy also relies on the expansion and *in vivo* persistence of CAR T cells. Both mFMC63-CAR4 T cells and Hu1E7-CAR4 T cells showed comparable proliferative abilities (**Figures 6A, B**). These results suggested that despite the lower binding ability of Hu1E7 scFv, Hu1E7-CAR4 T cells receive sufficient activation signal from the CAR molecule to initiate clonal expansion to that of mFMC63-CAR4 T cells. We also examined the cytokine secretion of both anti-CD19-CAR4 T cells. The results showed that mFMC63-CAR4 T cells secreted remarkably higher levels of IFN-γ and TNF-α than those of Hu1E7-CAR4 T cells (**Figures 6C, D**). Further studies in the kinetic binding properties of Hu1E7 scFv may provide a better understanding of the functional activities that may lead to additional improvement in Hu1E7-CAR4 T cells. The result of the cellular response of CAR T cells correlated with the different affinities of scFvs that were previously described (43, 44).

Cytokine-mediated toxicity is an important clinical problem that limits CAR T cell therapies (45). To mitigate this toxicity, a previous study (38) attempted to modify the hinge region of CD28-based CAR2 designs. The release of IFN-γ and TNF-α from T cells expressing CAR with the CD8α hinge and CD28 transmembrane (CD8-28Z) was significantly lower when T cells expressing CD28 hinge and CD28 transmembrane (28Z). Furthermore, this CAR design linked to a fully human scFv (Hu19-CD8-28Z) was evaluated in a clinical trial (46). T cells expressing Hu19-CD8-28Z demonstrated efficient anti-lymphoma activity with lower levels of neurologic toxicity and cytokine release syndrome (CRS) in the patients with B cell lymphoma (47). The results of our studies that used CAR4 containing the same CD8-CD28 design showed the lower levels of proinflammatory cytokine (IFN-γ and TNF-α) secretion *in vitro* are in concordance with those previously reported.

In conclusion, the new CAR T cells containing murine anti-CD19 (FMC63) scFv and three co-stimulatory domains linked to CD3ζ (28BB27ζ) (mFMC63-CAR4 T cells) were more efficient than mFMC63-CAR2 T cells in killing HeLa-CD19^+^ and Raji cells. Although the human anti-CD19 (Hu1E7) scFv showed relatively lower binding ability compared to that of the murine anti-CD19 (FMC63) scFv, Hu1E7-CAR4 T cells and mFMC63-CAR4 T cells demonstrated similar anti-tumor activities and cell proliferation. Hu1E7-CAR4 T cells secreted lower levels of cytokines (IFN-γ and TNF-α) than mFMC63-CAR4 T cells did. Further studies to compare the anti-tumor efficacy and safety of Hu1E7-CAR4 T cells and mFMC63-CAR4 T cells in animal models and clinical trials are required.

## Data Availability Statement

The raw data supporting the conclusions of this article will be made available by the authors, without undue reservation.

## Ethics Statement

The studies involving human participants were reviewed and approved by Siriraj Institutional Review Board (SIRB) of the Faculty of Medicine Siriraj Hospital, Mahidol University, Mahidol University (COA number: Si 762/2016). The patients/participants provided their written informed consent to participate in this study.

## Author Contributions

YW conceptualized and conducted the main experiments, collected and analyzed data, interpreted results and prepared manuscript. JS and PYe conceptualized and guided this research. JS, NS, PYo, KK, PYu, and CT partly performed experiments. NP and MY provided materials. JS, AP, TC, MJ, and PYe managed the study provided materials and reagents, and edited the manuscript. All authors contributed to the article and approved the submitted version.

## Funding

This work was financially supported by the National Science and Technology Development Agency (NSTDA) (grant no. P-16-50727), the International Research Network (IRN), and the Thailand Research Fund (TRF) (grant no. IRN58W0001), the Siriraj Research Fund of the Faculty of Medicine Siriraj Hospital (grant no. R016034008), and Mahidol University Grants (grant no. R016110006 and R016210017). YW was supported by an International Research Network (IRN)-Ph.D. Scholarship (IRN5801PHDW01). JS was supported by Mahidol University Grants and the Office of National Higher Education Science Research and Innovation Policy Council (NXPO) by Program Management Unit-Competitiveness (PMU-C) (grant no. C10F630063). MJ and PYe were supported by Siriraj Chalermphrakiat Grants. MY was supported by Thailand Science Research and Innovation (TSRI) (grant no. RTA6180012) and National Research Council of Thailand (grant no. NRCT 808/2563).

## Conflict of Interest

The authors declare that the research was conducted in the absence of any commercial or financial relationships that could be construed as a potential conflict of interest.

## Publisher’s Note

All claims expressed in this article are solely those of the authors and do not necessarily represent those of their affiliated organizations, or those of the publisher, the editors and the reviewers. Any product that may be evaluated in this article, or claim that may be made by its manufacturer, is not guaranteed or endorsed by the publisher.
