# Supplementary material for: Development of a Novel Anti-CD19 CAR Containing a Fully Human scFv and Three Costimulatory Domains
Source: Front Oncol. 2022 Jan 18;11:802876. doi: 10.3389/fonc.2021.802876 (PMC8804167; doi:10.3389/fonc.2021.802876)
Supplement: Supplementary file 1 [file DataSheet_1.docx]

***Supplementary Materials and Methods***

1. **Engineered HeLa and K562 cell lines overexpressing CD19**

HeLa and K562 cells, which normally have no expression of CD19, were engineered to overexpress CD19 on their cell surface so they could be used as surrogate target cells for the selection of human anti-CD19 scFv. The HeLa and K562 cells were transduced with a lentiviral construct containing human CD19 cDNA (NM_001178098.2). To engineer HeLa cells, 1x10^5^ cells were plated and cultured in a 5% CO_2_ humidified incubator at 37° Celcius (C) overnight. The supernatant containing lentiviruses harboring CD19 cDNA was added to the cells in the presence of 8 μg/ml polybrene and incubated at 37°C for 24 hours (h). The lentiviral supernatant was then removed, and transduced cells were selected using 0.5 μg/ml of puromycin to obtain a greater than 90% CD19^+^ population. To engineer K562 cells, 1x10^5^ cells were transduced with lentiviruses containing CD19 cDNA in the presence of 10 μg/ml protamine sulfate for 48 h. After removal of the lentiviral supernatant, cell surface expression of CD19 on transduced K562 cells was examined. The K562-CD19^+^ population was then stained with allophycocyanin APC conjugated anti-CD19 and sorted using a BD FACSAria III Cell Sorter flow cytometer (BD Biosciences, San Jose, CA, USA).

1. **Soluble human scFv protein expression in bacteria**

After biopanning, the eluted phages were infected into *E. coli* HB2151 (non-suppressor strain) for expression and screening of human anti-CD19 scFv phage clones. The transformed *E. coli* were cultured in M9ZB medium containing ampicillin (100 μg/mL) and glucose (1% w/v). When the optical density at 600 nm (OD600) of the culture reached 0.9, the cells were harvested and cultured at 30°C in glucose-free M9ZB containing 100 μg/mL ampicillin in the presence of isopropyl-β-D-thiogalactopyranoside (IPTG; Affymetrix, Santa Clara, CA, USA). The culture supernatant containing human anti-CD19 scFv protein from each clone was separated on a 12% polyacrylamide gel. After blotting, the human anti-CD19 scFv protein was detected using an anti-Myc mAb (clone 9E10; Santa Cruz Biotechnology, Dallas, TX, USA). The plasmid of selected phage clones that were able to secret soluble human anti-CD19 scFv were amplified for the human anti-CD19 scFv sequence using phagemid-specific primer pairs (**Supplementary Fig.6**).

1. **Soluble human anti-CD19 scFv protein expression in mammalian cells**

To study anti-CD19 scFv bioactivity (**Supplementary Fig.5**), human anti-CD19 scFv sequences were amplified from phagemid DNAs and cloned into a lentivirus vector with a C-terminal 6xHistidine tag sequence. Lentiviruses carrying human anti-CD19 scFv genes were prepared and transduced into Chinese Hamster Ovary (CHO)-K1 cells (Cat# CCL-61; ATCC). (CHO)-K1 cells were maintained in DMEM/F12 (Gibco; Thermo Fisher Scientific) supplemented with 10% FBS and 100 μg/ml of penicillin/streptomycin. Cell-free supernatant from CHO-K1 cells expressing stable human anti-CD19 scFv was prepared and used to purify histidine-tagged human anti-CD19 scFv protein via passage through TALON^®^ metal affinity chromatography columns (Takara Bio). The histidine-tagged anti-CD19 scFv protein was eluted with 500 mM imidazole (Sigma-Aldrich) and dialyzed in sterile PBS at 4°C overnight. The protein concentration was determined using Bradford dye reagent (Bio-Rad Laboratories, Hercules, CA, USA).

1. **Immunoblot analysis**

The CAR protein expression in HEK293T cells transfected with the transfer plasmid – pCDH.EF1α-scFv-CD19-CAR4 by Lipofectamine2000^®^ (Thermo Fisher Scientific) method, was examined by immunoblot analysis. After transfection for 48 h, the transfected HEK293T cells were harvested and lysed in 1% NP-40 lysis buffer. Proteins in the cell lysates were separated on 12% polyacrylamide gel and transferred to a nitrocellulose membrane. The full-length CAR protein on the membrane was probed with mouse anti-human CD3 zeta (CD3ζ) (clone E-3; Santa Cruz Biotechnology). The membrane was also probed with mouse polyclonal anti-human glyceraldehyde 3-phosphate dehydrogenase (GAPDH) antibody (Santa Cruz Biotechnology), which was used as a loading control. After washing 3 times with Tris-buffered saline (TBS) containing 0.1% of Tween 20 (TBST), the membrane was incubated with secondary antibody conjugated with horseradish peroxidase (HRP). The band of CAR protein was then detected by generation of chemiluminescent signals using SuperSignal™ Chemiluminescent Substrate (Thermo Fisher Scientific) and X-ray film exposure.

1. **Production of lentiviral particles**

Lenti-X^TM^ 293T cells were co-transfected with three plasmids, including transfer plasmid (pCDH.EF1α-scFv-CAR4), structural plasmid (psPAX2) containing HIV *gag-pol* genes, and envelope plasmid (pMD2.G) containing the *VSV-G* gene, at a ratio of 5:3.5:1, respectively, by calcium phosphate precipitation method. Supernatants containing lentiviruses were collected at 48- and 72-h post-transfection and filtered through a 0.45 μm filter membrane (MicroLab Science Co. Ltd., Zhejiang, China). The lentiviral particles were concentrated by high-speed centrifugation (J2-MC; Beckman Coulter, Inc., Brea, CA, USA) at 20,000 g at 4°C for 90 min. The concentrated viruses were stored in a -70°C freezer until use. The titer of lentivirus was determined using Lentivirus Titration Kit (Applied Biological Materials, Inc., Richmond, British Columbia, Canada).

1. **Immunophenotyping of T cells**

The fluorescence-labeled monoclonal antibodies used for immunophenotyping were anti-CD3-FITC (clone UCHT-1), anti-CD4-APC (clone MEM-241), anti-CD4-PerCP (clone MEM-241), anti-CD8-APC (clone UCHT-4), anti-CD19-APC (clone LT19), anti-CD16-APC (clone 3G8) (all Immunotools), and anti-CD56-PE (clone 5.H11) (BioLegend, San Diego, CA, USA). For analysis of states of T cell differentiation, the monoclonal antibodies used were CD45RO-FITC (clone UCHL-1) and CD62L-PE (clone HI62L) (Immunotools). For analysis of T cell exhaustion markers, the stained CD3^+^ T cells were co-stained with anti-PD1-PE (clone eBioJ105) (eBiosciences, San Diego, CA, USA), anti-LAG3-PE (clone 11c3c64), and anti-TIM3-PE (clone F38-2E2) (both BioLegend, San Diego, CA, USA). In brief, the cells were stained with the monoclonal antibodies at 4°C for 30 min in the dark. After washing 3 times, the cells were analyzed using a BD Accuri™ C6 Plus Flow Cytometer (BD Biosciences). Viable cells were gated according to their typical forward/side scatter characteristics and acquired at least 10,000 relevant events. The collected data was analyzed using BD Accuri™ C6 Plus software (BD Biosciences) and FlowJo 10 software (FlowJo LLC, Ashland, OR, USA).

**Table S1**. The number of clones obtained from human scFv phage display library screening to identify scFv binding specifically to the CD19 antigen.

| **Phage biopanning** | **Input titer**  **(CFU)** | **Output**  **(CFU*)** | **Ratio**  **(Output/Input)** | **Rescue phage**  **(CFU)** |
| --- | --- | --- | --- | --- |
| 1^st^ round of selection | 7x10^13^ | 3.96x10^4^ | 5.66x10^-10^ | 1.76x10^12^ |
| 2^nd^ round of selection | 1.76x10^12^ | 1.35x10^7^ | 7.67x10^-6^ | 2x10^11^ |
| 3^rd^ round of selection | 2x10^11^ | 9x10^4^ | 4.5x10^-9^ | 5.8x10^10^ |
| **scFv characterization** | **Input** | **Output** | **Frequency of positive clones** | |
| Expressed soluble scFv | 288 | 5 | 1.7% | |
| Different scFv clone | 5 | 5 | 100% | |
| scFv that can bind to CD19 | 5 | 1 | 20% | |

CFU is a colony-forming unit titrated in *E.coli* TG1, indicating the number of phage clones used in each selection round.

CFU* is a colony-forming unit titrated in *E.coli* HB2151, indicating the number of eluted bound phages obtained from each round of biopanning.

**Table S2.** Deduced amino acid sequences of VH and VL of Hu1E7 scFv obtained from DNA sequencing, and the results of analysis using the International Immunogenetics Information System (IMGT) Basic Local Alignment Search Tool (BLAST).

| **Clone** | **Family** | **CDR3** | **Germline** | **Amino acid differences from germline** |
| --- | --- | --- | --- | --- |
| **VH gene** |  |  |  |  |
| **Hu1E7** | **VH3** | **ARARTRTPAFDI** | **IGHV3-21*01** | **0** |
| **VL gene** |  |  |  |  |
| **Hu1E7** | **VL2** | **SYTSSSTFEV** | **IGLV2-14*01** | **13** |

**Table S3.**

| **% Identity of mFMC63 vs Hu1E7** | | | |
| --- | --- | --- | --- |
| **VH-FR1** | **54.17%** | **VL-FR1** | **36.00%** |
| **VH-CDR1** | **25.00%** | **VL-CDR1** | **33.33%** |
| **VH-FR2** | **52.94%** | **VL-FR2** | **47.06%** |
| **VH-CDR2** | **28.57%** | **VL-CDR2** | **33.33%** |
| **VH-FR3** | **48.57%** | **VL-FR3** | **55.56%** |
| **VH-CDR3** | **20.00%** | **VL-CDR3** | **50.00%** |
| **VH-FR4** | **90.00%** | **VL-FR4** | **50.00%** |
| **Total VH** | **50.85%** | **Total VL** | **41.51%** |

**
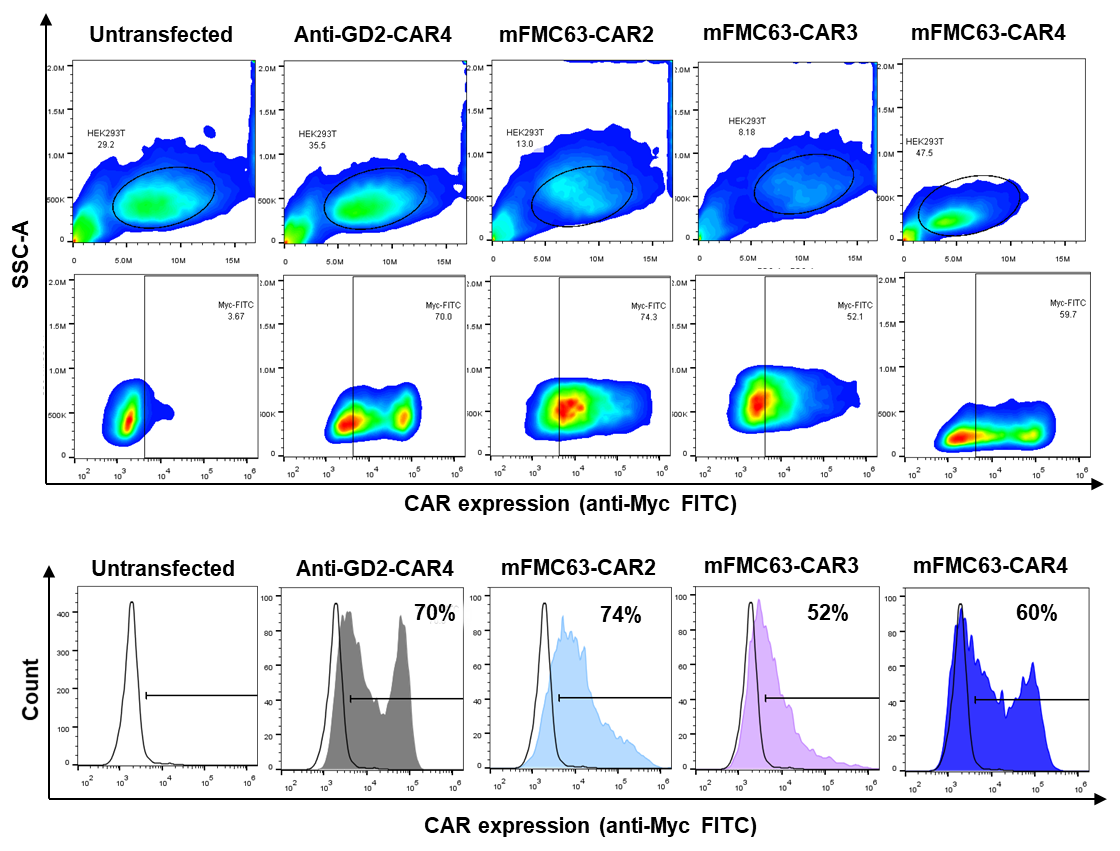
**

**Figure S1.** Psuedocolor plots are presented the gating strategy for detection of surface expression of anti-GD2-CAR4, mFMC63-CAR2, mFMC63-CAR3, and mFMC63-CAR4 proteins in transfected HEK293T cell lines. The cells were stained with chicken anti-Myc-FITC polyclonal antibody (FITC, fluorescein isothiocyanate).


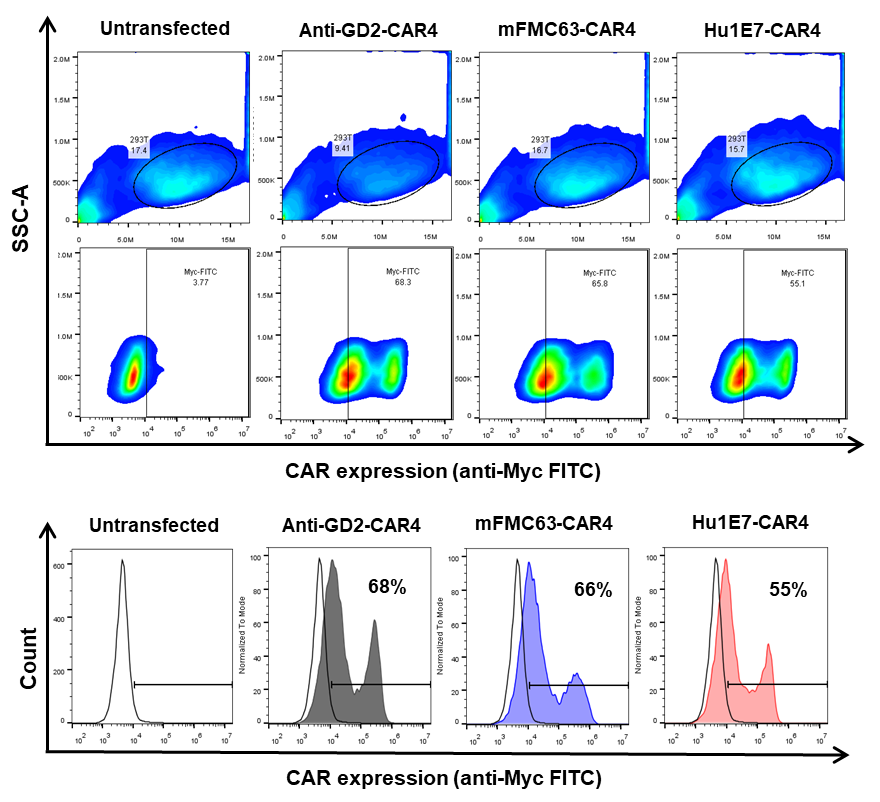


**Figure S2.** Psuedocolor plots are presented the gating strategy for detection of surface expression of anti-GD2-CAR4, mFMC63-CAR4, and Hu1E7-CAR4 proteins in transfected HEK293T cell lines. The cells were stained with chicken anti-Myc-FITC polyclonal antibody (FITC, fluorescein isothiocyanate).


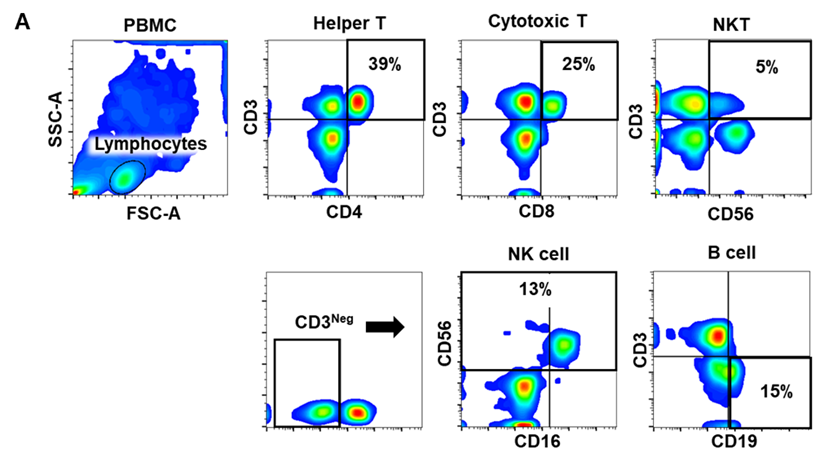


**Figure S3.** Characterization of scFv-CD19-CAR4 T immunophenotypes. Pseudocolor density plots show the gating strategy used to evaluate effector immune cell profiles of healthy individuals. The effector cell subsets is distinguished by CD3^+^CD4^+^ (helper T cells), CD3^+^CD8^+^ (cytotoxic T cells), CD3^Neg^CD56^+^CD16^+/Neg^ (NK cells), CD3^+^CD56^+^ (NKT cells), and CD3^Neg^CD19^+^ (B cells). Numbers indicate the percentages of cells in bold rectangle gate.


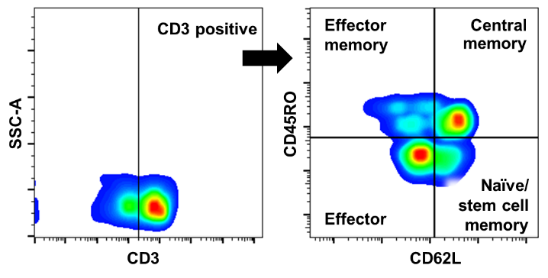


**Figure S4.** T cell differentiation states of scFv-CD19 CAR4 T cells. Flow cytometric analysis for the expression of the lymphoid-homing molecule CD62L, and the activation/effector marker CD45RO on CD3^+^ lymphocytes. The data shown reflect the gating of naïve (TN: CD45RO^Neg^/CD62L^+^), central memory (T_CM_: CD45RO^+^/CD62L^+^), effector memory (T_EM_: CD45RO^+^/CD62L^Neg^), and effector cells (T_EFF_: CD45RO^Neg^/CD62L^Neg^).


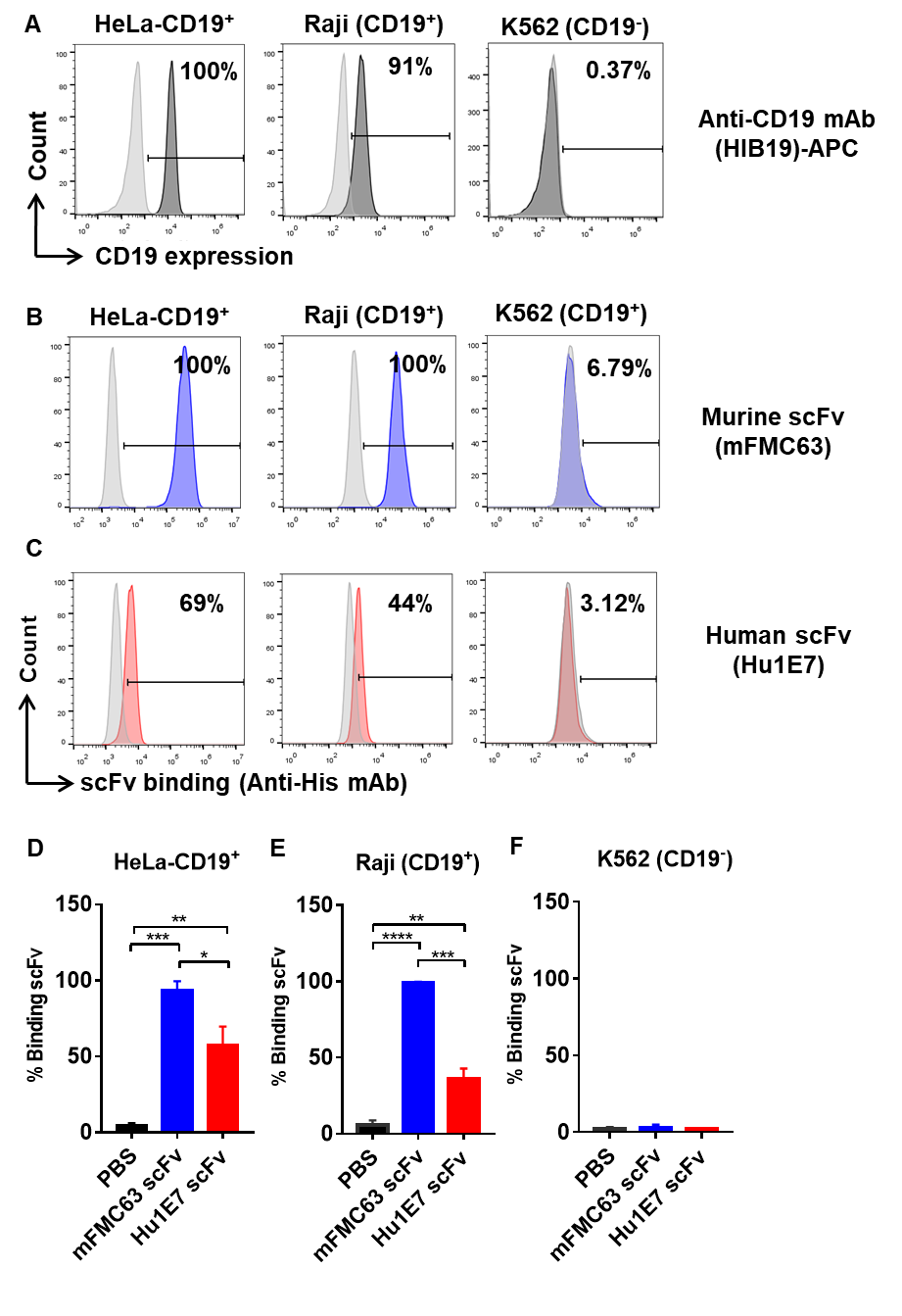


**Figure S5.** The binding abilities of the mFMC63 and Hu1E7 scFv proteins to CD19 antigen expressed on the cell surface of HeLa-CD19^+^ and Raji cells as analyzed by flow cytometry. (**A**) CD19 antigen expression on HeLa-CD19^+^ and Raji cells. K562, which showed no expression of CD19 antigen, was used as negative control. (**B**) The binding ability of the mFMC63 scFv protein to CD19 antigen expressed on HeLa-CD19^+^, Raji (CD19^+^), and K562 (CD19^‑^) cells. (**C**) The binding ability of the Hu1E7 scFv protein to CD19 antigen expressed on HeLa-CD19^+^, Raji (CD19^+^), and K562 (CD19^‑^) cells.


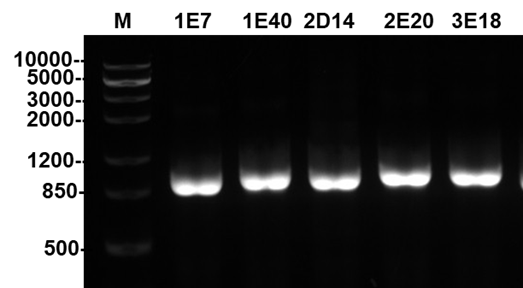


**Figure S6.** DNA inserts amplified by polymerase chain reaction (PCR) of 5 bacteriophage clones containing human scFv binding specifically to CD19 that were selected by screening with HeLa CD19^+^ cells.
